# Continuous selection pressure to improve temperature acclimation of *Tisochrysis lutea*

**DOI:** 10.1371/journal.pone.0183547

**Published:** 2017-09-13

**Authors:** Hubert Bonnefond, Ghjuvan Grimaud, Judith Rumin, Gaël Bougaran, Amélie Talec, Manon Gachelin, Marc Boutoute, Eric Pruvost, Olivier Bernard, Antoine Sciandra

**Affiliations:** 1 Sorbonne Universités, UPMC Univ Paris 06, CNRS-INSU, Laboratoire d'Océanographie de Villefranche-sur-mer (LOV), Villefranche-sur-mer, France; 2 INRIA BIOCORE, Sophia Antipolis Cedex, France; 3 IFREMER, PBA, Nantes, France; International Centre for Genetic Engineering and Biotechnology, INDIA

## Abstract

Temperature plays a key role in outdoor industrial cultivation of microalgae. Improving the thermal tolerance of microalgae to both daily and seasonal temperature fluctuations can thus contribute to increase their annual productivity. A long term selection experiment was carried out to increase the thermal niche (temperature range for which the growth is possible) of a neutral lipid overproducing strain of *Tisochrysis lutea*. The experimental protocol consisted to submit cells to daily variations of temperature for 7 months. The stress intensity, defined as the amplitude of daily temperature variations, was progressively increased along successive selection cycles. Only the amplitude of the temperature variations were increased, the daily average temperature was kept constant along the experiment. This protocol resulted in a thermal niche increase by 3°C (+16.5%), with an enhancement by 9% of the maximal growth rate. The selection process also affected *T*. *lutea* physiology, with a feature generally observed for ‘cold-temperature’ type of adaptation. The amount of total and neutral lipids was significantly increased, and eventually productivity was increased by 34%. This seven month selection experiment, carried out in a highly dynamic environment, challenges some of the hypotheses classically advanced to explain the temperature response of microalgae.

## Introduction

Microalgae are a promising source of resources and bulk chemicals for various applications such as nutrition, aquaculture, cosmetics and, at a longer time scale, biofuels or green chemistry. However, the wild type strains commonly cultivated offer poor industrial yields, limiting their profitability. These wild species adapted to natural conditions do not tolerate extreme temperatures reached by artificial culturing systems (photobioreactors or greenhouse cultures) with a low thermal inertia [1][2]. Moreover, in temperate climates, microalgae production throughout the year is limited by the low winter temperatures. This sensitivity to temperature therefore restricts production, unless temperature is regulated with a high cooling/heating system involving high financial and environmental costs [1]. The need to select new strains adapted to a wider range of temperature is thus crucial for increasing productivity and reducing cultivation costs.

The steady state growth response of microalgae to temperature, i.e. the growth rate as a function of temperature in acclimated and non-limiting conditions, also called the thermal reaction norm, has been extensively studied [2][3], and can be accurately predicted by the model of [4]. This model is parameterized by three cardinal temperatures: T_min_ and T_max_, corresponding respectively to the temperatures below and above which growth is not possible. T_opt_, the optimum growth temperature, was defined as the temperature leading to the highest growth rate (all other parameters being kept constant). The temperature values ranging between T_min_ and T_max_ represent the thermal niche. The selection of strains with a larger thermal niche and thus lower temperature sensitivity is a promising approach to increase outdoor productivity.

Microalgal strains acclimate to temperature in a process similar to photoacclimation [5]. Under weak thermal perturbation and on short term exposition (less than 10 generations), acclimation enhances ecological fitness by modulating the flux of energy produced by photosynthesis to carbon fixation rate through the Calvin cycle in a more efficient way [6]. However, when the thermal stress exceeds its physiological tolerance, adaptation mechanisms are triggered. The genetic modifications triggered during the adaptation process result from the selection of individuals with highest fitness in the new thermal stressing conditions. Adaptation occurs by two different mechanisms: selection of the most adapted individuals among the pre-existing genetic diversity of a non-monoclonal population (pre-selective mutations), and *de novo* genetic mutations induced by environmental stress (adaptive mutation) [7]. In this paper “adapted strain” was defined as the resulting strain supporting temperature stresses that the simply “acclimated strain” do not tolerate. As it is shown later, adaption also results in a different lipid profile, compared to the initial strain, when submitted to the same environment. Little information exists on physiological temperature adaptation in microalgae. The only available studies are for extremophile strains, resulting of a million year evolutionary process of thermal adaptation. Life at low temperatures has selected individuals that could mitigate the lowering of their metabolic activities and growth by synthetizing psychrophilic enzymes with higher activity at cold temperatures [8][9] or stimulating enzyme production [10][11], and can preserve the fluidity of their membranes by increasing the synthesis of unsaturated fatty acids [12][13][3]. Conversely, under high temperature conditions that tend to denature proteins and membranes [10][14], extremophile adapted microalgal species produce more Heat Shock Proteins [15] or saturated fatty acids, allowing them to resist these lethal effects [14]. This study therefore investigated the possibility of changing the temperature response (growth rate and physiology) of a non-extremophile strain of *Tisochrysis lutea*, keeping this species for several months in continuous cultures, under increasing stressful thermal conditions consisting in daily temperature shifts. This species is well-used in aquaculture and mollusk hatcheries for fish larva and bivalve nutrition especially because of its high content in essential polyunsaturated fatty acids. It is a fast growing microalgae (μ_max_ about 2 d^-1^), easy to cultivate under temperate climate, requiring a pH about 7, a solar irradiance of 780 μmol photons.m^-2^.s^-1^ at about 30°C [16].

## Materials and methods

### Microalgae strain

The *Tisochrysis lutea* strain (CCAP 927/17) used in this experiment, named W2X, was obtained by a selection/mutation process from the original strain CCAP 926/14. This strain is characterized by a doubled triglyceride productivity [17][18]. Note that the strain used was non-monoclonal and thus contained a pre-existing genetic diversity supposed to facilitate the emergence of new traits.

### Culturing system

The culturing system was specifically designed to maintain long-term continuous cultures of microalgae in computer-controlled growing conditions. The enrichment medium was prepared in several 20 L tanks (Nalgene) filled with 3 weeks-matured natural seawater, previously filtered on 0.1 μm, and autoclaved at 120°C for 40 min. After cooling, f/2 medium [19] was added through a 0.22 μm sterile filter. The culture vessels consisted of water jacketed, 1.9-liter plane photobioreactors (thereafter named “selectiostat”) connected to a circulating programmable water bath (Lauda Proline RP845). 30 to 40 min were necessary to vary the temperature from 10 to 40°C inside the selectiostats. Cultures were continuously and gently homogenized by a magnetic stirrer and bubbling air. They were illuminated with LEDs (Nichia NVSL219BT 2 700°K) placed on one side of the photobioreactors. The light intensity, continuously measured with a probe (SKY, SKL2620) placed on the opposed side of the reactor was maintained at I_in_ = 250 μmol photons.m^-2^.s^-1^. pH was maintained constant at 8.2 by computer-controlled microinjections of CO_2_. Turbidity was measured on-line using a red LED (Rodan HIRL5010) at 800 nm on one side of the reactor and a photodiode (Optek OP993) on the other side. Light, pH, temperature, turbidity, and dilution rate were continuously monitored by ODIN^®^ software [20].

### Cleaning procedure

As the selection experiment lasted about 260 days in stressing conditions, algae biofilm was removed monthly. After saving 1 liter of culture in an autoclaved Schott bottle, the different pieces of the selectiostats were dismounted and washed with milliQ water then 70% ethanol. Once dried and reassembled, the photobioreactors were sterilized with 10% HCl and then rinsed with fresh sterile medium filtered through 0.22 μm (SpiralCap, Gelman). Selectiostats were then inoculated with the preserved culture, and complemented with sterile medium filtered through 0.22 μm with a Stepdos pump (KNF) to readjust the culture volume.

### Turbidostats vs fedbatch modes

Two selectiostats were processed in parallel, each of them using a different culturing mode. In the selectiostat named STurb (turbidostat mode), the dilution rate was dynamically changed by the ODIN^®^ software through a PID control algorithm to maintained turbidity at a constant level corresponding approximately to a cell concentration of 9 10^5^ cell.mL^-1^.

The biomass set point was chosen as the maximal value permitting to avoid marked light shading. The nitrogen concentration in the inflowing medium permitted a biomass concentration of 3.3 10^6^ cell.mL^-1^. Moreover for cell concentration higher than 5 10^6^ cell.mL^-1^, light limitation was observed in the selectiostat. To be conservative, a biomass concentration below 10^6^ cell.mL^-1^ was chosen to fulfill these conditions.

In the selectiostat named SFb (fed-batch mode), a fraction of the culture volume was replaced with fresh sterile medium every 7 days. Only 5 to 10% of the initial volume was kept, in order to restart cultures with an initial cell density of 5 10^5^ cell.L^-1^.

### Continuous selection procedure, the “ratchet” protocol

Our selection method was inspired from the original protocols proposed by [16] and [17], but instead of gradually increasing the culture temperature, the daily average temperature was kept unchanged at 28°C along the experiment (Eq 1). The stress consisted in progressively increasing the amplitude of the daily temperature variation pattern. Indeed, square wave temperature variations were applied, with 8 hours at low temperature (T_low_) followed by 16 hours at high temperature (T_high_) with an average daily temperature of 28°C.

$$T_{daily\;average}=\;\frac{1}{24}\;\left(T_{high}\;x\;16+\;T_{low}\;x\;8\right)$$ (1)

**Fig 1:**
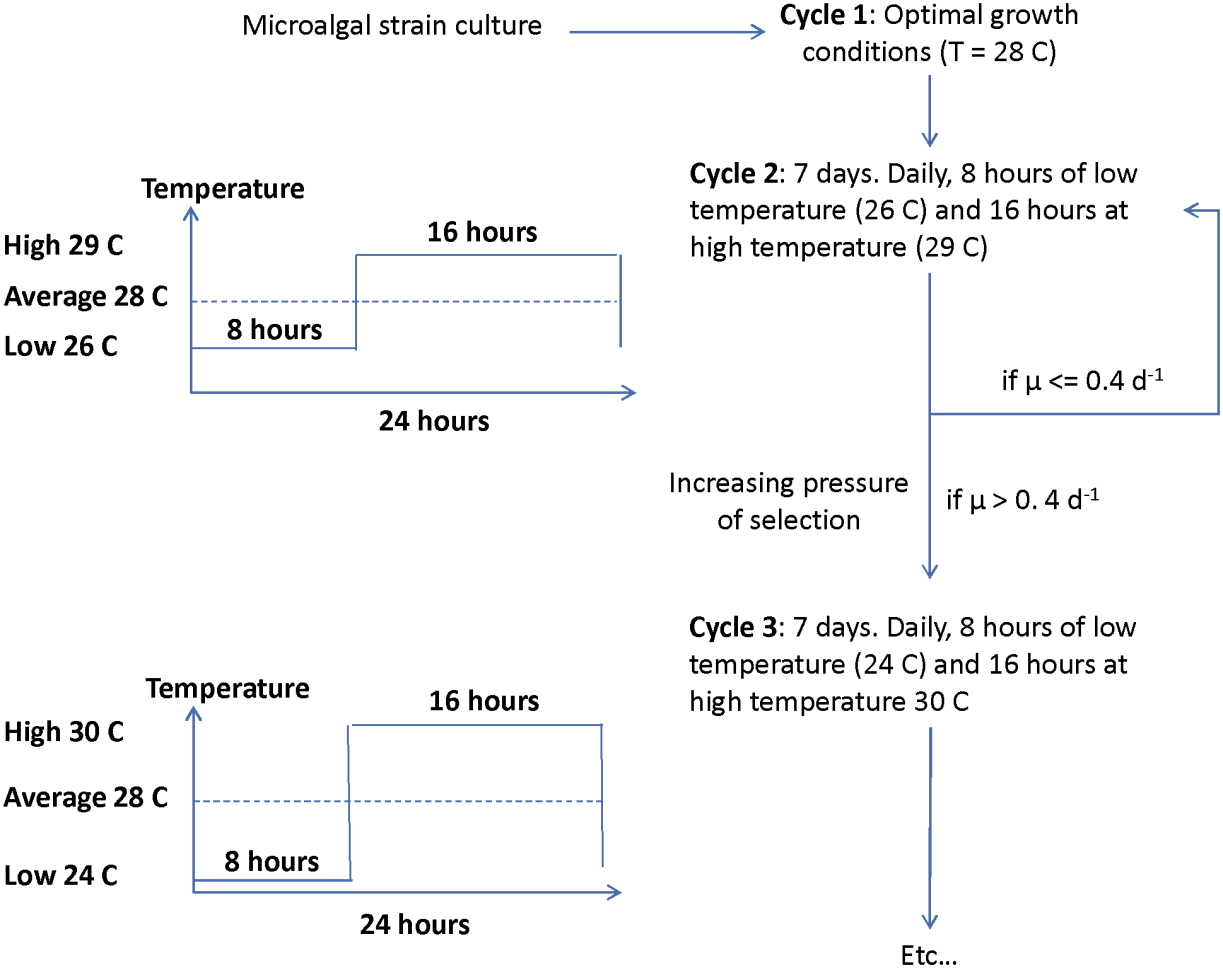
Temperature conditions applied during the 3 first cycles of the selection experiment in STurb and SFb. The same protocol with increasing temperature amplitudes was used for the following cycles (4 to 10).

**Fig 2:**
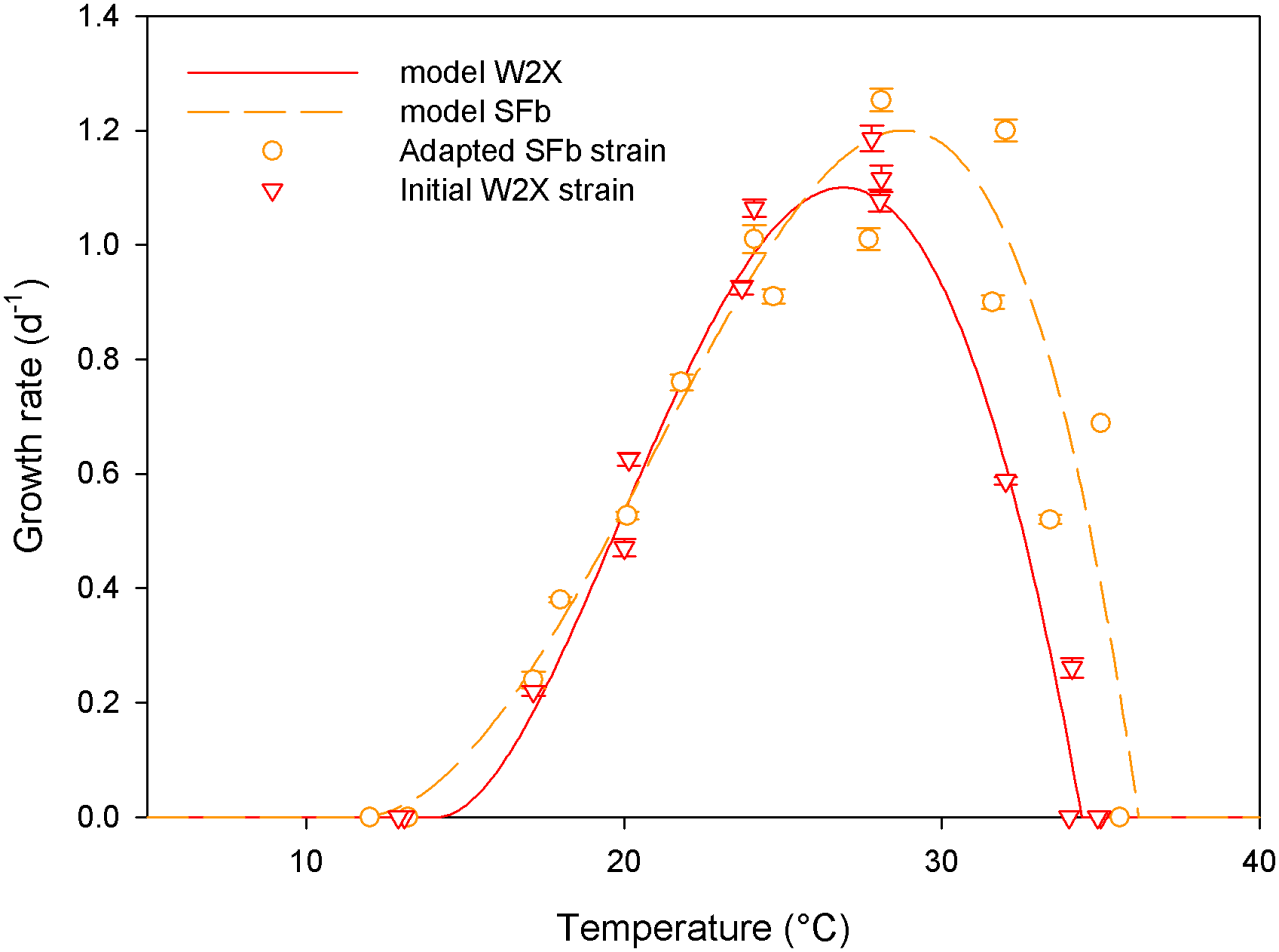
Growth rate measured at different temperatures in the TIP device for the initial W2X strain (triangles) and the temperature selected strain SFb (open circles). Lines represent the best fit of the [4] model to the data series.

This daily pattern was repeated identically over 7 days (i.e. a selection cycle). This duration allowed to avoid nitrogen starvation in the fed-batch mode and to reach equilibrium in the turbidostat mode. At the end of a selection cycle, if the measured growth rate was higher than 0.4 d^-1^ (i.e. more than 50% of the maximum growth rate, meaning that cells were able to cope with temperature stress), a new cycle was implemented by reducing T_low_ by 2°C and increasing T_high_ by 1°C. Otherwise the temperature conditions were remained unchanged to provide a longer adaptation period (Fig 1). The rationale behind this asymmetrical mode was motivated by the asymmetry of microalgal response to temperature, where the variation of the growth rate observed for temperatures higher than T_opt_ is generally steeper than below T_opt_ (Fig 2) [4]. For the two last selection cycles 9 and 10, T_high_ was increased by only 0.5°C and T_low_ decreased by only 1°C in order to reduce the mortality resulting from the very large daily variations of temperature at the end of the experiment (Table 1).

**Table 1:** Temperature conditions applied during the selection experiment. A cycle was a set of temperature conditions (T_min_ and T_max_) daily applied to culture during selection procedure.

# cycle	T_Low_ (°C)	T_High_ (°C)	Number of feed batches	Cycle duration (d)
1	28.0	28.0	6	42
2	26.0	29.0	1	7
3	24.0	30.0	1	7
4	22.0	31.0	2	14
5	20.0	32.0	2	14
6	18.0	33.0	2	14
7	16.0	34.0	1	7
8	14.0	35.0	2	14
9	13.0	35.5	23	161
10	12.0	36.0	3	23

### Cell population

Size distribution of the cell population was monitored once or twice a day with an optical particle counter (HIAC—Royco; Pacific Scientific Instruments). Mean cell diameter and biovolume were calculated from the size distribution. The variability between triplicate samples was routinely lower than 5%. The first and the second counting were performed at the beginning of the low and high temperature periods, respectively. Measurements were performed at least once per cycle.

The average growth rate, experimentally measured for a cycle of selection ${\bar{\mu }}_{Exp-SFb}$ (d^-1^) of the SFb culture was calculated using the following equation:

$${\bar{\mu }}_{Exp-SFb}=\frac{Ln\left(\frac{BV_{2}}{BV_{1}}\right)}{t_{2}-t_{1}}$$ (2)

where BV_1_ and BV_2_ were the biovolume (μm^3^.mL^-1^) at time *t*_1_ and *t*_2_ (d), respectively. The growth rate was the average of 4 days of measurements (n > 4). The computation of the average growth rate in a continuous culture (${\bar{\mu }}_{Exp-STurb}$) with permanent stress affecting growth is tricky and would have needed high frequency biomass measurements. For the routine daily monitoring of the selectiostats, we used the average dilution rate over one week. However, this proxy of average growth rate turned out not to be accurate enough to support the discussion (standard deviation of measurements > 10%). Therefore, the evolution of the growth rate for the turbidostat (which qualitatively follows a similar trend as for the fed batch) was not used for the analyses, and only the features of the final adapted strain are discussed in this paper.

### Growth response with respect to temperature (TIP experiment)

A special device (TIP) composed with 17 photobioreactors (0.5 L) and permitting to control independently temperature, pH and light intensity [16] was used to assess the temperature response on growth at constant pH and irradiance [16]. Three strains were used, *T*. *lutea* before (W2X strain, stored at 19°C for 2 years) and after (STurb and SFb, stored for one month at 21°C) the selection experiment. After one day acclimation in the TIP, the exponential growth rate was estimated at 8 temperatures ranging from 12°C to 35.5°C from the linear regression of the logarithm of DO_680_ (n>12). Irradiance (250 μmol.m^-2^.s^-1^) and pH (7.9) were maintained constant. Cell concentration was assessed by image analysis after Lugol staining on Malassez slides using specific software (Samba Technologies, Meylan, France) to check the DO_680_ measurement validity. Lipid classes and fatty acid composition were analyzed for STurb and SFb strains growing at 28°C after 7.2 days of cultivation in nitrogen starvation and compared with the measurements obtained during the W2X lipid comparison experiment (detail below).

### Characterisation of temperature response

The three cardinal temperatures (T_min_, T_max_ and T_opt_) were estimated for the initial W2X and for the new selected strains SFb and STurb, obtained after 10 months of selection, by fitting the model proposed by [4] to the TIP growth response (Fig 2). A gradient-based optimisation procedure was run and a jackknife analysis provided an estimate of these parameters together with their confidence intervals [4].

To quantify the effect of temperature selection on the measured growth rate of SFb strain, a “selection coefficient” *s*_*SFb*_ [21] was calculated as follow:

$$s_{SFb}\;=\text{ ln}({\bar{\mu }}_{Exp-SFb})-\text{ln}({\bar{\mu }}_{Th-W2X})$$ (3)

where ${\bar{\mu }}_{Th-W2X}$ is the theoretical growth rate of the strain W2X calculated for the same temperature conditions as experienced by the culture SFb during each selection cycle, with the cardinal parameter of the W2X strain:

$${\bar{\mu }}_{Th-W2X}\left(T_{low},T_{high}\right)=\frac{7.5}{24}\mu_{W2X}\left(T_{low}\right)+\;\frac{15.5}{24}\mu_{W2X}\left(T_{high}\right)+\;\frac{1}{24}\mu_{W2X}\left(T_{average}\right)$$ (4)

where *T*_*low*_ and *T*_*high*_ are the low and high temperature values set during the selection cycles, and *T*_*average*_ the mean temperature experienced during the 1h transition time between low and high temperatures. Selection occurs if the frequency of adapted strains in the population increases (s > 0) [21].

### W2X lipid comparison experiment (benchmark experiment)

To compare the lipid content of the initial W2X strain with the new adapted strains, a benchmarking experiment in similar conditions than the TIP experiment was performed. Three cylindrical glass photobioreactors of 2 L were used as triplicates. Temperature was controlled at 28°C and pH maintained constant at 7.9 by micro-additions of CO_2_ in the bubbling air. Continuous light was provided by fluorescent tubes (Dulux^®^1, 2G11, 55W/12-950, lumilux de lux, daylight, OsramSylvania) at 250 μmol.m^-2^.s^-1^ and measured in the center of the empty photobioreactors as in the TIP device. Cultures were gently homogenized by magnetic stirring. The enrichment medium was prepared in several 20 L tanks (Nalgene) filled with 3 weeks-matured natural seawater, previously filtered on 0.1 μm, and autoclaved at 120°C for 40 min. After cooling, f/2 medium was added through a 0.22 μm sterile filter [19]. Nitrogen concentration was lowered at f/4 concentration to reach N starvation. Lipid sampling (400 mL of culture) was realized at the end of the experiment during the starvation phase.

### Cellular content analysis

6.65 mL triplicates of culture were sampled weekly, 3 days after the beginning of the selection cycle and just after the temperature shift from T_high_ to T_Low_, and filtered onto glass-fiber filters (Whatman GF/C, threshold 1.2 μm) precombusted at 450°C for 12h. For particulate carbon and nitrogen measurement, samples were kept at 60°C until analyses were performed with a CHN analyzer (2400 Series II CHNS/O, Perkin Elmer). For pigments, samples were kept at -80°C, and extracted in acetone (3 ml) for 1 hour at 4°C in the dark with frequent and gentle stirring. After 5 min of centrifugation (JOUAN G 412) at 2000 rpm, supernatant was analyzed with a spectrophotometer (Perkin Elmer UV/Vis Spectrophotometer Lambda2). Chlorophyll *a*, *b* and total carotenoid were determined using the equations of Lichtenthaler and absorbance at 470.0, 644.8 and 661.6 nm [22]. The variability between triplicates was lower than 7%.

For adapted strains, lipid samplings were made at the end of the TIP experiment, after 7.7 days of cultivation in nitrogen starved conditions at 28°C. For the W2X, lipids were sampled at the end of the lipid comparison experiment (6.9 days) in N-starved conditions. Known volumes of culture were centrifuged (JOUAN G 412) for 10 min at 2000 rpm, after the addition of 100 μL of Cl_3_Fe (50 mg.mL^-1^) as flocculate. The lipid extraction protocol was derived from Bligh and Dyer [23]. Total lipids were determined gravimetrically. Neutral lipids, glycolipids and phospholipids were separated on Extract-Clean SPE Silica 500 mg/8 mL (Alltech) and eluted respectively with 6 column volumes of chloroform, 4 column volumes of acetone and 6 column volumes of methanol completed with 2 volumes of methanol–10% ammonia. Total lipids were placed on top of the column in chloroform. All fractions were dried under vacuum and weighted to provide an estimate of each class of lipids.

The lipid productivity (μg_Lipid_.mL^-1^.d^-1^) of the different strains during the benchmarking experiment was calculated as follows:

$$P_{lipid}=\frac{Lipid_{f}-Lipid_{i}}{t_{f}-t_{i}}$$ (5)

where *Lipid*_*f*_ and *Lipid*_*i*_ are the total lipid concentration (μg.mL^-1^) at final and initial time respectively. Note that, the initial lipid concentration was not measured (not enough matter for our lipid protocol). Initial lipid content per gram carbon was thus assumed to be the same as the final content. This hypothesis provides a slightly underestimated productivity, but has limited impact since initial biomass is very low. Fatty acid analysis began by converting saponifiable lipids into methyl esters with 7% boron trifluoride in methanol [24]. Gas chromatography (GC) of fatty acid methyl esters (FAME) was carried out on a 30 m length 0.32 mm internal diameter quartz capillary column coated with famewax (Restek) in a Perkin-Elmer XL autolab GC, equipped with a flame ionization detector (FID). The column operated isothermally at 185°C. Helium was used as carrier gas at 7 psig. Injector and detector were maintained at 250°C. The variability was routinely 3% for major components, 1–9% for intermediate components and 25% for minor components (<0.5% of total fatty acids). Measurements were relative and expressed as percentage of total fatty acid, no internal standard was needed.

## Results and discussion

### From acclimation to adaptation

**Fig 3:**
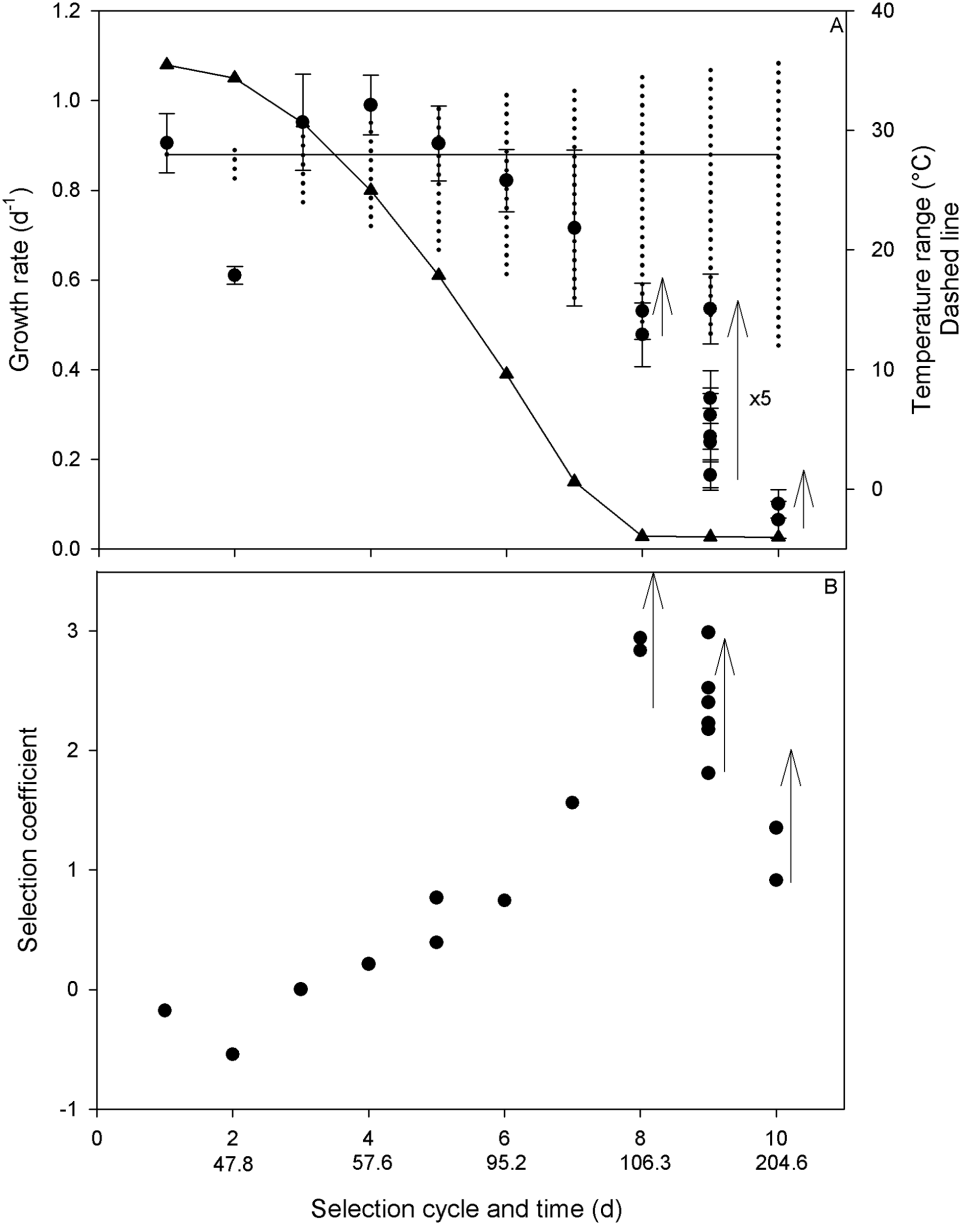
A) Mean growth rates measured for SFb strain during each selection cycle (black circles), and theoretical growth rate calculated with the calibrated temperature model of the initial strain W2X (triangles) submitted to the same temperature conditions. Growth rate was measured at a minimum of 6 different dates on one culture (SFb, n>6), during the exponential phase to achieve a linear regression with error < 5% and thus an accurate growth rate determination. The vertical dashed lines indicate the amplitude of temperature variations during each cycle, and the horizontal continuous line corresponds to the mean temperature maintained constant at 28°C throughout the selection experiment. B) Time change of the selection coefficient measured in SFb. When measured during consecutive weeks of the same cycle, the selection coefficient increased with time (vertical arrows).

Fig 3A shows that the theoretical growth rate of the initial strain W2X calculated for the experimental temperature conditions (Eq 4), decreased with the amplitude of temperature variations. Fig 3A also shows that, except for the very first 2 cycles, the growth rates measured in the SFb selectiostat were significantly higher than the theoretical growth rate computed for the parameters of the initial strain. It is also noticeable that, for the temperature conditions of cycles 8, 9 and 10, the growth is still possible in the SFb selectiostat, while no growth could be observed at these temperatures for the initial strain. The reason why the growth rates in the SFb selectiostat measured during the two first cycles were lower than the theoretical growth rate for the initial W2X strain might be due to an initial acclimation phase to the variable temperature conditions. It is likely that, by maintaining the cells for a longer time in the temperature conditions of cycles 1 and 2, the growth rate would have progressively increased as a result of a combination of acclimation and adaptation. This increase in average growth can indeed clearly be observed for the last cycles which were repeated several weeks. As a matter of illustration, the average growth rate during the cycle 9 was multiplied by 5 when comparing the first and the last week of the cycle (Fig 3A). The selection coefficient ***s*** (Fig 3B) was positive after cycle 3; this means that the frequency of new thermally adapted individuals in the population increased to the detriment of the initial strain [21]. This increase shows that the initial strain was not fitted to these highly changing temperature conditions. From cycle 8 to 10 (T_Low_ = 12°C; T_High_ = 35.5°C), the transition from a cycle to the next led systematically to a reduction of the selection coefficient. After the transition in the new cycle, it gradually increased with the number of generations highlighting the progressive emergence of cells better fitted to the selective pressure (Fig 3B; black arrows).

With a Luria-Delbrück fluctuation analysis [7], Costas et al. [25] studied the variance of the fitness during selection conditions and determined the origin of mutant adapted to the new thermal conditions. They increased the average growth temperature as a stressing parameter for *Isochrysis galbana* wild type (T_max_ = 28°C). Under a low temperature range (15–30°C), the increase of the strain fitness was due to acclimation or pre-selective mutations (selection from the pre-existing genetic diversity). Under a higher range (30–35°C), they assumed that the observed response was linked to adaptive mutations (occurrence and selection of new mutants). With our protocol, it was not possible to identify the source of fitness increase. Since the pressure of selection was dynamic, in contrast to [25], the observed selection was probably a dynamical equilibrium involving acclimation, pre-selective mutations and adaptive mutation to the emergence of a new population with a broader thermal niche and/or a higher thermal acclimation capacity.

### New strain characterization

**Fig 4:**
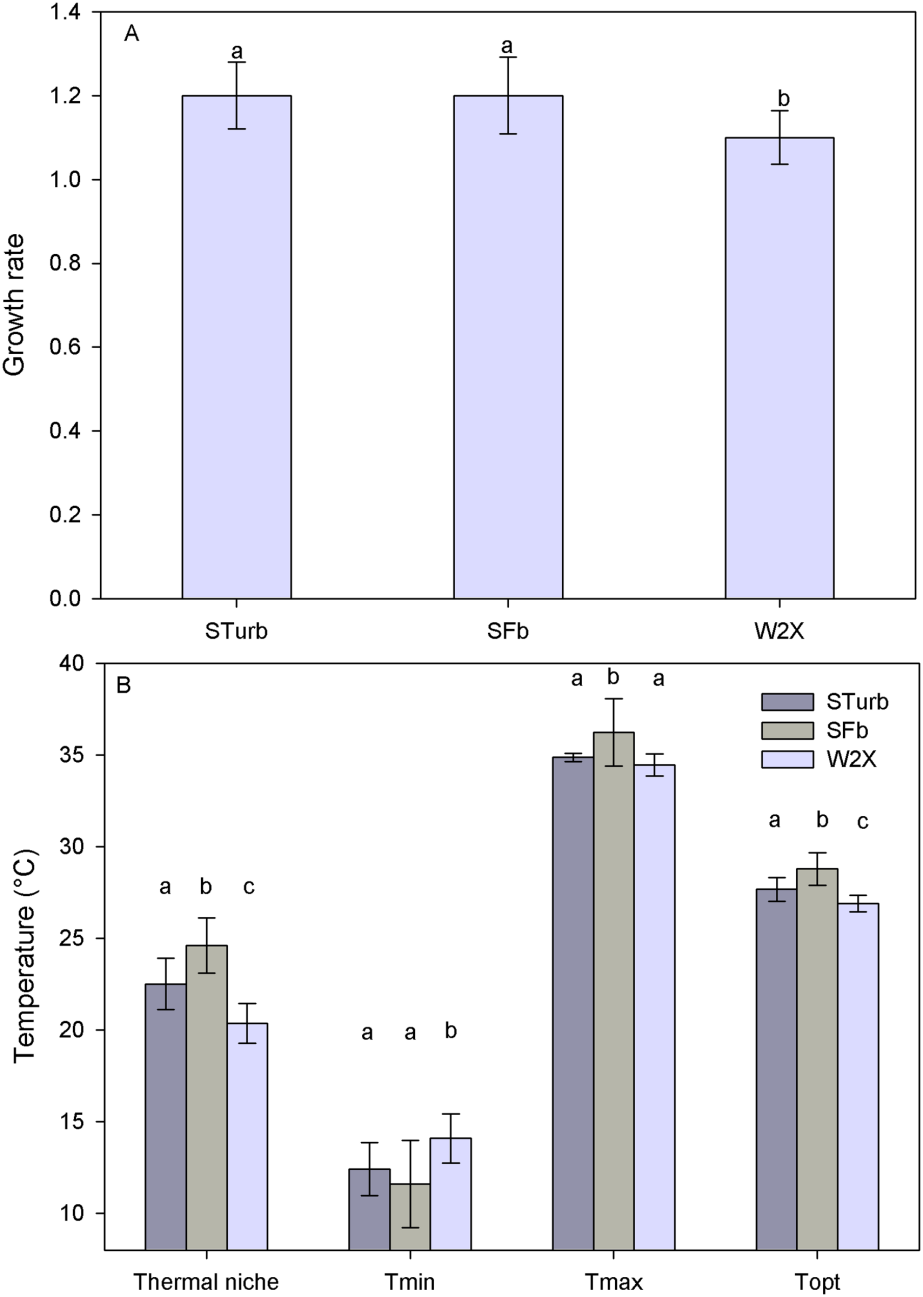
Comparison between the new STurb and SFb strains and the initial W2X strain. A) Growth rate. B) Cardinal temperatures and thermal niche. Values are expressed as the mean ± standard deviation determined by a jackknife analysis. Groups marked with the same letter (a, b or c) are not statistically different (1% error, Student test). Different letters indicate groups statistically different.

The new strains were obtained after 41 generations in fed-batch (SFb) and approximately 157 generations in turbidostat (STurb; value obtained from the estimate of the average growth rate of the STurb population based on the dilution rate). The thermal response of these two final new strains and the initial strain W2X was characterized with the TIP [16] and compared (Fig 2). The cardinal temperatures for these new strains were assessed using the model of [4]. The optimal growth rates of the two new selected strains were significantly higher than the rate of the initial W2X (+9%; statistical difference at 1%, Student test; Fig 4A). This result was expected because it was demonstrated mathematically and experimentally that continuous medium renewal eliminates preferentially individuals that grow slower, leading to an increase of the population mean growth rate [26][27]. Consistent with Eppley [28], the increase in the optimal growth rate was correlated with an increase in T_opt_ (Fig 4). However, despite the “hotter is faster” trend [29], the Eppley curve normalized by the optimal growth rate of the W2X strain overestimates the evolution of the optimal growth rate by 41.7% and 51.1% for STurb and SFb respectively.

The trade-off between thermal specialist and thermal generalist strains (narrow thermal niche/high performances and large thermal niche/low performances respectively) is a common hypothesis in literature. A microorganism with an increased thermal niche should have a lower maximal growth rate. This assumption has sometimes been mathematically transcribed by a constant area beneath the temperature response growth curve [29][30][31]. However, this widespread assumption in evolutionary models of thermal adaptation is hardly supported by experimental data for microorganisms. Bennett & Lenski [32] have refuted this assumption since they recorded, after 2000 generations, an increasing T_opt_ of some *E*. *coli* strains without modifying the thermal niche. To our knowledge, our experiment with microalgae is the first to assess this assumption. The increase in growth rate coincided with an increase in the thermal niche (+11% and +22% respectively for final STurb and SFb strains; statistical difference 1%, Student test; Fig 4). The dynamic environment used in our experiments is probably a key factor explaining the inconsistency of the trade-off theory for *T*. *lutea*. The thermal niche increase was the consequence of two significant effects, an increase in T_max_ and a decrease in T_min_ (Fig 4). When comparing the initial W2X thermal niche (20°) to the thermal niche of other microalgal species (30°C for S*cenedesmus sp*, 34°C for *Dunaliella tertiolecta*, 24°C for *Porphyridium cruentum* [4]), it appeared that the initial strain was probably a thermal specialist. On the other hand, the new adapted strains with a thermal niche close to 25°C were closer to thermal generalists.

Previous studies carried out with bacteria have shown that these microorganisms adapt their optimal growth temperature (T_opt_) to the average temperature of their environment [33] or slightly above as hypothesized by some models [34] but very few data reinforce this hypothesis. After an adaptation of three months at constant average temperatures, [11] recorded the same growth rate for *Skeletonema costatum at* 3°C and 18°C. Likewise, by submitting *Isochrysis galbana* to increasing constant average temperatures, [35] reported positive growth at 35°C for the adapted strain, whereas the wild type did not grow at this temperature. Here, T_opt_ of the new strains increased during the selection process and was at the end higher than the average temperature (28°C). This evolution is consistent with [36] who compared the T_opt_ modelled for 195 species with the average temperature of the species location.

Our experiment, carried out with intense daily temperatures variations but constant mean value, highlighted that temperature adaptation is complex and depends not only on the average temperature but also on the temperature range and its dynamics. It also highlighted the importance to consider all the cardinal temperatures and not only the optimal growth temperature as it is too often the case.

### Evolution of the biochemical composition across the selection procedure

In *T*. *lutea*, nitrogen starvation increases the total lipid content [37][38]. The initial W2X strain (CCAP 927/17), produces 2 times more neutral lipids (1.7 times more total lipids and a higher ratio of neutral to polar lipids) than the wild type (CCAP 927/14; [17]). After our thermal selection experiment, it was necessary to check the preservation of these highly interesting lipid properties. Lipid content of the adapted strains cultivated at 28°C during the TIP experiment was compared with measurements made on the initial W2X, during the benchmark experiment, in the same culture conditions. Starvation state were similar (C:N ratio: 20.9, 17.7 and 18.0 ± 1.3 mol:mol respectively for SFb, STurb and W2X).

**Fig 5:**
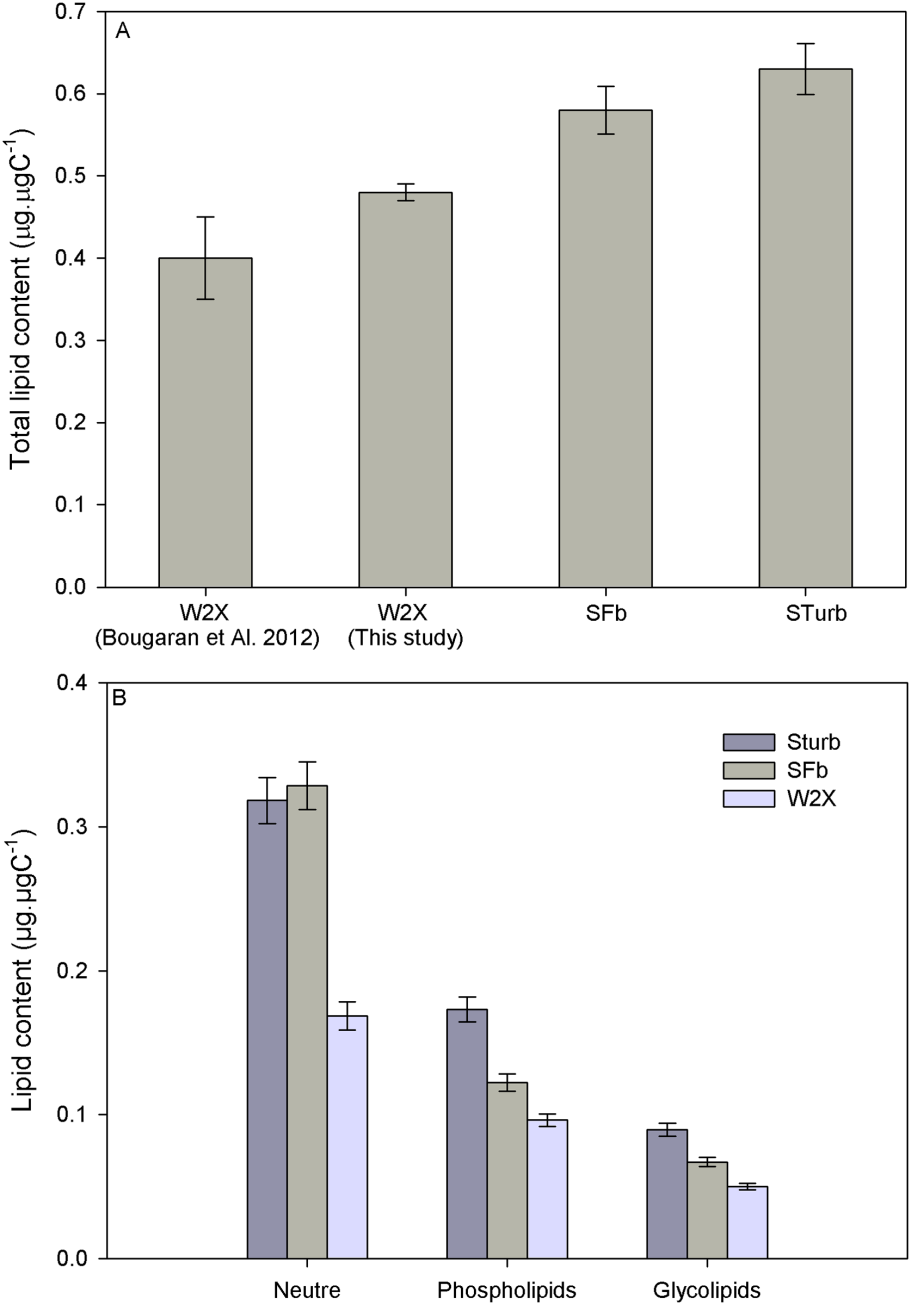
Comparison of the total lipids (A) and lipid classes (B) between the adapted Sturb and SFb strains (n = 3; measurement triplicates) and the initial W2X strain (n = 3; independent culture triplicates) after nitrogen starvation.

The adapted SFb and STurb strains had higher total lipid content than the initial W2X strain (0.58, 0.63 and 0.48 ± 0.01 μg.μgC^-1^ respectively for SFb, STurb and W2X; Fig 5A). Lipid content found for the W2X was in accordance with result from [17] for the same strain (0.4 ± 0.05 μg.μgC^-1^). It could be hypothesized that total lipids were enhanced due to the thermal stress response induced by temperature variations. The acclimation response of total lipids to temperature is not clear in the literature [39]. For some species, total lipids increased as temperature decreases, and reach maximum levels at extreme temperatures [40][40][41][3]. Other authors found a strong link between total lipid content and growth rate [42][43][14]. However, only acclimation to temperature was studied without emphasis on long term evolution.

Moreover, the repartition in lipid classes changed strongly between the adapted and the initial strains. Under nitrogen starvation, adapted strains exhibited higher neutral lipid content (+ 92%) and higher phospholipid content (+53%) than the W2X strain (Fig 5B). No difference was observed in the relative repartition of free fatty acids between these strains (Table 2). In response to cold temperatures, membrane cell fluidity is preserved by an increase of polyunsaturated fatty acid and a decrease of saturated and monosaturated fatty acid [14]. Very few information exist on lipid class response to temperature (phospholipids, glycolipids and neutral lipids). Some works suggest that an increase of the temperature increases total lipids and neutral lipids [44]. Once again, these works studied only acclimation. Lipid adaptation to temperature was never experimented. Lipids were affected by the thermal selection procedure but the attractive lipid profile of the initial strain in term of neutral lipids production was not degraded but on the contrary improved. At the end, total lipid productivity of the adapted strains was increased by 34% when compared with the initial W2X (13.6, 14.1 and 10.3 μg_Lipid_.mL^-1^.d^-1^ for Sturb, SFb and W2X respectively).

**Table 2:** Relative fatty acid composition of the two new adapted strains in comparison with the initial W2X strain (n = 3; independent culture triplicates) as a % of total fatty acid.

	W2X	Standard deviation	STurb	SFb
Saturated				
ISO14:0	0.02	0.01	0.11	0.02
C14:0	23.64	0.88	27.95	23.92
ISO15:0	0.30	0.02	0.35	0.22
ANT15:0	0.14	0.02	0.13	0.15
C15:0	0.36	0.03	0.36	0.26
ISO16:0	0.04	0.01	0.14	0.08
ISO17:0	0.16	0.00	0.08	0.07
ANT17:0	0.04	0.01	0.05	0.04
C16:0	16.06	0.18	15.08	16.02
C17:0	0.08	0.00	0.05	0.05
C18:0	0.57	0.08	0.35	0.54
Total	41.86	0.89	44.64	41.37
Mono-ene				
C14:1n-5	0.71	0.08	0.78	0.62
C15:1n-8	0.03	0.01	0.03	0.03
C15:1n-6	0.01	0.00	0.02	0.02
C16:1n-7	2.98	0.23	2.93	2.26
C16:1n-5	0.32	0.01	0.34	0.46
C18:1n-9	22.27	1.14	18.03	24.37
C18:1n-7	2.94	0.24	0.96	1.43
C18:1n-5	0.45	0.05	1.32	0.53
C20:1n-9	0.10	0.01	0.07	0.04
C20:1n-7	0.03	0.00	0.00	0.04
C20:1n-5	0.08	0.01		
C22:1n-13+11	0.22	0.06	0.25	0.31
C22:1n-9	0.12	0.16	0.27	0.41
Total	30.35	0.81	24.99	30.51
Di-ene				
C16:2n-6	0.14	0.01	0.08	0.07
C16:2n-4	0.41	0.02	0.34	0.26
C18:2n-6	4.14	0.15	3.85	4.79
C20:2n-6	0.16	0.03	0.55	0.17
Total	5.32	0.13	4.82	5.29
Tri-ene				
C16:3n-4	0.05	0.01	0.02	0.01
C16:3n-3	0.23	0.01	0.20	0.17
C18:3n-6	0.20	0.02	0.11	0.11
C18:3n-3	2.25	0.08	4.05	4.75
C20:3n-6	0.13	0.03	0.10	0.09
C20:3n-3	0.05	0.00	0.12	0.12
Total	2.95	0.11	4.60	5.24
Tetra-ene				
C16:4n-3	0.02	0.00	0.05	0.01
C16:4n-1	0.04	0.00	0.08	0.06
C18:4n-3	5.88	0.29	7.18	6.18
C20:4n-6	0.24	0.01	0.14	0.18
C20:4n-3	0.08	0.00	0.08	0.05
C22:4n-6	0.14	0.01	0.20	0.06
Total	6.48	0.27	7.72	6.53
Penta-ene				
C18:5n-3	1.27	0.08	0.35	0.22
C20:5n-3	0.24	0.01	0.23	0.23
C21:5n-3	0.43	0.03	0.24	0.14
C22:5n-6	1.68	0.06	1.98	1.92
C22:5n-3	0.27	0.22	0.11	0.06
Total	3.89	0.26	2.90	2.58
C22:6n-3	9.14	0.72	10.32	8.48
Total PUFA	27.78	0.28	30.36	28.11

**Fig 6:**
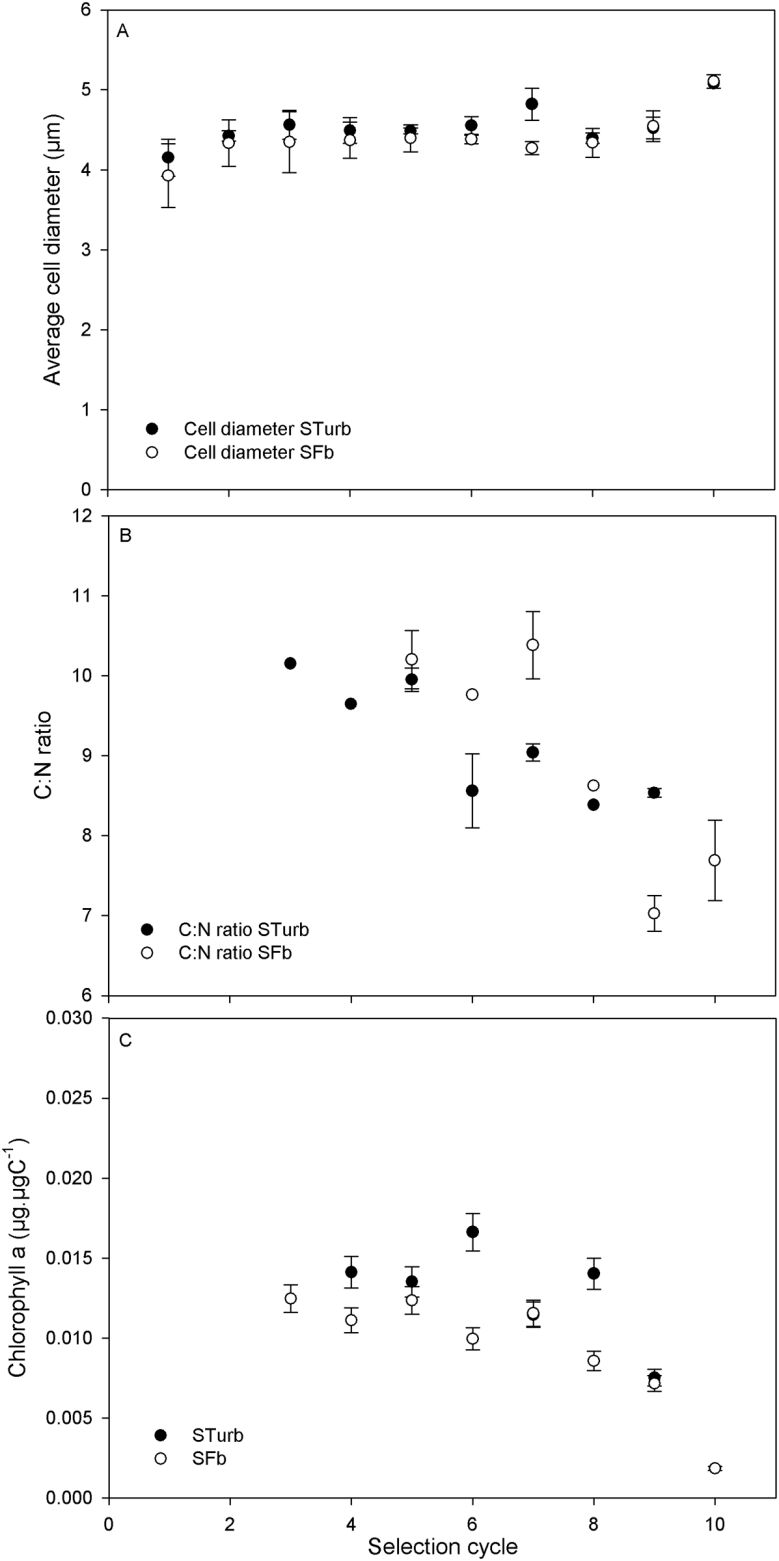
Evolution of three biological markers during the selection process at the first iteration of each cycle. A) Evolution of the cell diameter. B) C: N ratio C) Chl a: Carbon ratio. Standard deviation was calculated on triplicate of independent measurements.

It is generally observed that warmer temperatures are more favorable to smaller cells, probably due to the increase in enzymatic reaction rates [45][46]. During this selection procedure, the average cell diameter did not show statistical differences (ANOVA, p-value > 0.1). However, for the last selection cycles (9, 10), a cell diameter increase was observed (Fig 6A). Moreover, the C:N ratio gradually decreased (Fig 6B), while the Chl a:C ratio started to decrease after selection cycle 6 (Fig 6C). All these observations are characteristic of cold adaptation pattern, as reported by [3] and [47] for *T*. *lutea*. This acclimation features at lower temperature reflect the reallocation of cell resources (energy, carbon) necessary to enhance the temperature-dependent biochemical reactions of the photosynthesis dark phase and to rebalance the energy produced by the temperature-independent photochemical reactions involved in the photosynthesis light phase [47].

These results were unexpected since the daily average temperature was constant throughout the selection (28°C). Our selection protocol turned out to be more selective towards cold temperatures than high temperatures, probably because the decrease in temperature at each selection cycle was more severe than its increase (-2°C and +1°C respectively). Secondly, this selection protocol was dynamic and necessitated a continuous thermal acclimation. This particular physiological status may offer a higher plasticity and acclimation capacity.

## Conclusion

Dynamical selection pressure in continuous culture, for long periods of time has a strong potential for domesticating strains for industrial applications. This study highlights the possibility to impact the thermal niche of *T*. *lutea* by modifying T_min_ and T_max_ with fluctuating temperatures but at constant daily average temperature. The increase in the thermal niche was also concomitant to an increase in the maximum growth rate and associated to important physiological changes similar to a ‘cold-temperature’ acclimation. This approach permitted to keep the initial lipid properties of the strains in term of neutral lipids production. One of the most interesting features triggered by the highly dynamic protocol must still be explored: the capacity of the new strains to acclimate more rapidly under large temperature fluctuations.
